# Growth performance and reproductive function impairment of glyphosate‐based herbicide in male guinea pig (*Cavia porcellus*)

**DOI:** 10.1002/vms3.443

**Published:** 2021-02-08

**Authors:** Valence Bwana Mutwedu, Albert Wafula Nyongesa, Pascaline Ciza Azine, Daniel Katulanya Chiregereza, Victor Herman Ngoumtsop, Yannick Mugumaarhahama, Rodrigue Basengere Balthazar Ayagirwe

**Affiliations:** ^1^ Department of Animal Production Faculty of Agriculture and Environmental Studies Université Evangélique en Afrique (UEA) Bukavu DR Congo; ^2^ Department of Veterinary Anatomy and Physiology University of Nairobi Nairobi Kenya; ^3^ Institute of Fisheries and Aquatic Sciences at Yabassi Douala Cameroon

**Keywords:** guinea pig, oxidative stress, seminal parameters, toxicity, WILLOSATE

## Abstract

Glyphosate formulations, widely applied non‐selective systemic herbicides, are progressively becoming the most controversial pesticides on the market due the adverse effects they pose to humans and environment. The information on these potential hazardous effects to the handlers of the pesticide remains obscure. This study investigated effects of glyphosate‐based herbicide on growth performance, seminal parameters and hemato‐biochemical profiles in male guinea pig. Forty sexually mature male guinea pigs weighing between 393.3 and 418.4 g were divided into four groups of 10 animals each and orally administered 0, 186, 280 and 560 mg/kg body weight of WILLOSATE daily for 60 days. Daily feed intake and body weight gain were recorded. At the end of experimental period all animals were humanely sacrificed, and blood samples and vital organs were collected for appropriate analysis. Results showed a significant decrease (*p* < 0.05) in body weight gain (−102.2%), final body weight (−9.8%) and feed intake (−13.1%) of animals following sub‐chronic exposure of WILLOSATE. The weights of the liver and kidney increased significantly (*p* < 0.05) by 25.4% and 28.8%, respectively, while testicular weights decreased (*p* < 0.05) by 24% with increasing doses of WILLOSATE. A decrease in sperm motility (−67.9%), viability (−52.7%) and concentration per vas deferens (−40.7%), and an increase in sperm major (28.1%) and minor (45.3%) morphological aberrations were recorded in WILLOSATE – exposed guinea pigs when compared to controls. There was a dose‐dependent increase (*p* < 0.05) in MCV and WBC and a decrease in Hb content and RBC, as well as serum content in total protein (−11.8%). The serum content of cholesterol (37.8%), urea (87.1%), creatinine (22.4%), ALAT (74.2%) and ASAT (88.7%) were significantly higher in treated groups compared to controls. These results point toward the toxic effects of WILLOSATE on vital organs and reproductive function of the body at high doses and long‐term exposure.

## Graphical abstract

This study investigated effects of glyphosate‐based herbicide (WILLOSATE) on growth performance, seminal parameters and haemato‐biochemical profiles in male guinea pig. Forty sexually mature male guinea pigs weighing between 393.3 and 418.4 g were divided into four groups of 10 animals each and orally administered 0, 186, 280 and 560 mg/kg b.w. of WILLOSATE daily for 60 days. These results point towards the toxic effects of GBH on vital organs and reproductive function of the body at high doses and at long‐term exposure.

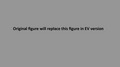

## 1 INTRODUCTION

Agriculture is one of the key sectors of the Democratic Republic of Congo's economy. Its role on the national Gross Domestic Product remains important, despite the excessive dependence on the mining sector. The large population growth in this country in the last decade necessitated farmers to intensify their agricultural production, resulting in a high incidence of plant diseases, pest and weed (World Bank, 2019). In response to this situation, a large number of chemicals such as pesticides have been used to increase or maintain agricultural productivity (Erisman et al., 2008).

Pesticides are chemicals used in agriculture to eliminate pests in order to improve the short term quantity and yield of agricultural production (Grasiela et al., 2014). However, because of their toxicological properties, ubiquity, persistence, presence and concentration in the food chain, they are responsible for genotoxicity (Benbrook, 2016) and fertility problems such as impairment of spermatogenesis, decreased motility and viability of spermatozoa in male (Ngoula et al., 2017) and disruption of oestrus cycle in females (Al‐Hamdani & Yajurvedi, 2017) exposed to contaminated food.

Glyphosate‐based herbicides (GBH) are among the non‐selective glyphosate formulations with a broad‐spectrum activity that was introduced in agricultural production fields in 1974 (Benbrook, 2016).

The annual use of GBH has increased substantially in the last two decades and it has been reported as the top ranked herbicide in several developed countries (Myers et al., 2016; Steinmann et al., 2012). The GBH is considered the most commonly used herbicide in Africa (Gianessi & Williams, 2011). However, it is slowly and progressively becoming significantly controversial pesticide product ever produced due to its toxicity effects (Gill et al., 2017; Owagboriaye et al., 2017). According to Bohn et al. (2013) and the European Food Safety Authority (EFSA, 2016), glyphosate residues had been detected in foodstuffs and drinking water polluted by rain surface runoff, thereby increasing routes of exposure in animals and humans.

It has been reported to exert its toxic effects via oxidative stress mechanism in animals by producing reactive oxygen species (ROS) (De Liz et al., 2013). Oxidative, haematological and biochemical damage have been observed in mice exposed daily to 50 and 500 mg/kg of ROUNDUP® (Jasper et al., 2012). In related studies, the chronic exposure of rats to ROUNDUP® resulted in kidney and liver damages with potential significant health implications for animal and human populations (Mesnage et al., 2015, 2017).

In Eastern part of DR Congo, most of livestock species (cattle, goat, pig, rabbit, guinea pig) are reared under traditional management and mostly fed with crop residues and food crop weeds (Akilimali et al., 2018; Metre et al., 2019; Mutwedu et al., 2015; Wasso et al., 2018), which poses potential danger to animals if forages were exposed to glyphosate‐based herbicides.

Several studies have already been conducted on glyphosate based herbicides (Gill et al., 2017; Jasper et al., 2012; Owagboriaye et al., 2017) but few have focused on WILLOSATE. However, many authors have already established a differential toxicity between GBH that would be related not only to glyphosate but also to adjuvant molecules. It is therefore important to establish the effects of WILLOSATE toxicity on male reproduction. In order to ascertain the insights of WILLOSATE on growth and reproductive function, the present study was designed to assess growth performances, live body weight gain, weights and volumes of organs, sperm characteristics, haematological and biochemical parameters of guinea pigs sub‐chronically exposed to varying doses of WILLOSATE.

## 2 MATERIALS AND METHODS

### 2.1 Study site, animal housing and feeding

The animals were marked at the ear for identification and housed in wooden cages (1 m × 1 m × 0.5 m) at room temperature with animal house relative humidity of 74 ± 6% and lighting conditions of 12 hr: 12 hr light: dark cycle during the trial period.

### 2.2 Chemical

The GBH used was WILLOSATE^®^ in a commercial formulation of glyphosate 36% (360 g/L), obtained from the local market. It was stored in its original container and kept in a cool, dry and well‐ventilated area at room temperature throughout the experiment.

### 2.3 Experimental design

After an acclimatization period of 2 weeks, animals were randomly divided into four groups of 10 guinea pigs each and orally administered with 10 ml/kg of distilled water for group 1 as control; 186, 280 and 560 mg/kg body weight of WILLOSATE, respectively, for groups 2, 3 and 4 during 60 consecutive days. The GBH was chosen since it is the most readily available herbicide on the market in this region and so commonly used but there is little awareness on its harmful effects to the user when not handled with care. The doses used represent 1/30, 1/20 and 1/10 of 5,600 mg/kg body weight considered as LD_50_ on rats (Monsanto Company, 1985; National Library of Medicine, 1992) and correspond to 0; 67; 103 and 202 mg/kg of glyphosate, which are in the range of developmental toxicity studies in laboratory animals (Williams et al., 2012). Water and feed were provided ad libitum. The WILLOSATE was diluted in a watery suspension and 5ml administered via oral gavage to the guinea pigs once a day for 60 consecutive days. During the trial period, feed intake (F.I.) and body weight gain (W.G.) were measured weekly.

### 2.4 Blood and organ sampling

At the end of the experimental period, all guinea pigs from each group were fasted for 24 hr and humanely slaughtered. The blood was collected directly via cardiac puncture before sacrificing. After sacrifice, the kidney, lung, heart, liver, testes, epididymis, vas deferens were collected by dissection, freed of adipose tissue, washed using saline solution and blot‐dried for weight and volume measurement. The relative weights of the organs were expressed as percentage of slaughter weights. Blood for haematological analysis was collected in a test tube with EDTA for analysis of haemoglobin (Hb), white blood cells (WBCs), red blood cells (RBCs), lymphocytes (LYM), mean cell volume (MCV), mean corpuscular haemoglobin (MCH), mean corpuscular haemoglobin concentration (MCHC), packed cell volume (PCV) using automated hematimeter MINDARY BC 3,000. Blood for biochemical analysis was collected in tubes free from anticoagulant, stored at room temperature and after 24 hr, the serum was collected and preserved at −20°C for the evaluation of total cholesterol, albumin, aspartate aminotransferase (ASAT), alanine aminotransferase (ALAT), urea, creatinine, protein and glucose, using CHRONOLAB® commercial kits (CHRONOLAB, Ref: 101‐0576).

### 2.5 Sperm characteristics

The harvested epididymis of each animal was carefully trimmed‐off fat, minced in 10 ml of warm 0.9% NaCl solution (37°C), and spermatozoa were obtained from cauda part of the epididymis following technique described by Sharma et al. (2009). Sperm motility was assessed using 20 μl of the warm 0.9% NaCl solution at ×40 magnification and the motility score was assessed according to the method described by Mohammed and Engidawork (2011). Sperm viability, expressed as percentage of swollen sperm, was analyzed using hypo‐osmotic swelling test (Amorim et al., 2009). For the morphology, the percentage of abnormal sperm was analyzed using Eosin/Nigrosin test. Five microliters of sperm were mixed with 5μl of Eosin/Nigrosin solution and morphological defects of head (major anomalies), mid‐piece, tail, and the proportions (minor anomalies) of cells affected were evaluated. For each of both parameters (viability and morphology), a total of 200 spermatozoa were counted in at least five different microscopic fields following the protocol of Revell and Mrod (1994). The sperm concentration was determined using Thoma hemacytometer cell.

### 2.6 Statistical analysis

All data were analyzed using XL STAT for Windows 10 Software. Results were expressed as mean ± *SD*, and treatment effects on observed parameters among experimental groups and controls were assessed using one‐way ANOVA. The statistical model applied for the analysis of variance was: Yij = μ + βi + εij where Yij is the observed value of the jth observation having received the ith treatment; μ is the mean of the observation in the studied population; βi is the effect of ith treatment and εij is the residual error associated to the observation j which received the treatment i. The differences in mean values of the observed parameter were compared using the Tukey HSD post hoc test at 5% significance level.

## 3 RESULTS

### 3.1 Growth performances

The final body weight and the body weight gain decreased (*p* < 0.05) with increasing dose of WILLOSATE (Table 1). However, no significant effect was observed on the average weekly feed intake.

**TABLE 1:** Effects of different levels of WILLOSATE on some growth performances in male guinea pigs

Parameters (*n* = 10)	Dose of WILLOSATE (mg/kg bw)				
	0	186	280	560	ER (%)
Initial body weight (g)	396.30 ± 48.98	393.80 ± 40.86	393.30 ± 34.72	418.40 ± 18.15	5.60
Final body weight (g)	461.80 ± 12.94^a^	430.60 ± 7.66^b^	424.40 ± 9.82^bc^	416.60 ± 5.12^c^	−9.80
Body weight gain (g)	65.50 ± 16.08^a^	36.80 ± 15.59^ab^	31.10 ± 18.81^ab^	−1.80 ± 21.19^b^	−102.20
Average weekly feed intake (g)	173.00 ± 11.60	176.00 ± 10.90	156.00 ± 14.80	153.00 ± 14.20	−13.10

Note

a,b,c: means with different letters are significantly different at *p* < 0.05, ER, effect rate; bw, body weight.

### 3.2 Relative weights and volumes of some organs

The relative weights of kidney and liver as well as the volume of liver increased (*p* < 0.05) in a dose dependent manner. The opposite trend was observed in the relative weight and volume of testis. No significant effect was observed on lung, heart, epididymis and vas deferens relative weights (Table 2).

**TABLE 2:** Effects of different levels of WILLOSATE on the relative weights and volumes of some organs in male guinea pigs

Parameters (*n* = 10)	Dose of WILLOSATE (mg/kg bw)				
	0	186	280	560	ER (%)
Weights organs (g)					
Kidney	0.52 ± 0.02^c^	0.57 ± 0.02^b^	0.57 ± 0.03^b^	0.67 ± 0.034^a^	28.80
Liver	2.01 ± 0.26^b^	2.01 ± 0.25^b^	2.28 ± 0.07^ab^	2.52 ± 0.05^a^	25.40
Lung	0.13 ± 0.03	0.13 ± 0.03	0.12 ± 0.04	0.15 ± 0.02	2.00
Heart	0.05 ± 0.03	0.04 ± 0.01	0.05 ± 0.01	0.05 ± 0.02	0.00
Testis	0.50 ± 0.03^a^	0.49 ± 0.03^a^	0.41 ± 0.03^b^	0.38 ± 0.03^b^	−24.00
Epididymis	0.10 ± 0.01	0.12 ± 0.01	0.12 ± 0.03	0.13 ± 0.03	3.00
Vas deferens	0.06 ± 0.01	0.06 ± 0.05	0.07 ± 0.01	0.06 ± 0.03	0.00
Volumes of organs (ml)					
Kidney	1.90 ± 0.15	1.85 ± 0.19	1.82 ± 0.18	1.85 ± 0.18	−5.00
Liver	10.50 ± 1.05^c^	11.16 ± 1.16^bc^	12.83 ± 0.98^ab^	14.00 ± 1.78^a^	33.30
Testis	1.25 ± 0.22^a^	1.21 ± 0.21^ab^	1.17 ± 0.13^ab^	0.93 ± 0.17^b^	−32.00

Note

a, b, c: means with different letters are significantly different at *p* < 0.05. ER, effect rate; bw, body weight.

### 3.3 Sperm characteristics

WILLOSATE administration to guinea pigs decreased sperm motility, viability and concentration (*p* < 0.05) in dose‐dependent manner compared to controls. The opposite trend was recorded with the percentages of major and minor anomalies (Table 3).

**TABLE 3:** Effects of different levels of WILLOSATE on sperm characteristics in male guinea pigs

Parameters (*n* = 10)	Dose of WILLOSATE (mg/kg bw)				
	0	186	280	560	ER (%)
Motility (%)	93.33 ± 10.32^a^	50.00 ± 16.73^b^	50.0 ± 20.97^b^	30.0 ± 10.95^b^	−67.90
Viability (%)	74.66 ± 9.68^a^	66.71 ± 4.82^a^	42.46 ± 4.97^b^	35.33 ± 4.03^b^	−52.70
Sperm concentration (10^6^/ml)	276.00 ± 68.14^a^	242.3 ± 41.59^a^	197.3 ± 48.04^b^	163.6 ± 56.12^b^	−40.70
Sperm morphology (%)					
Major anomalies (%)	7.96 ± 1.12^b^	7.5 ± 1.19^b^	11.48 ± 3.42^a^	10.20 ± 1.05^a^	28.10
Minor anomalies (%)	24.70 ± 12.38^ab^	19.73 ± 6.11^b^	35.58 ± 7.38^a^	35.90 ± 3.69^ab^	45.30

Note

a, b, c: means with different letters are significantly different at *p* < 0.05, ER, effect rate; bw, body weight.

### 3.4 Haematological parameters

WILLOSATE administration significantly decreased (*p* < 0.05) the Hb and RBC while total WBC and MCV increased when compared to controls. The other blood parameters were not significantly affected by WILLOSATE at all doses (Table 4).

**TABLE 4:** Effects of different dose levels of WILLOSATE on haematological parameters in male guinea pig

Parameters (*n* = 10)	Dose of WILLOSATE (mg/kg bw)				
	0	186	280	560	ER (%)
Hb (g/dl)	15.64 ± 1.031^a^	14.88 ± 0.77^ab^	14.03 ± 0.29^b^	14.80 ± 0.33^ab^	−5.70
PCV (%)	40.22 ± 1.84	38.73 ± 0.74	41.68 ± 4.69	42.66 ± 2.60	6.00
RBC (×10^12^/l)	5.78 ± 0.28^a^	5.62 ± 0.23^ab^	5.29 ± 0.14^b^	5.40 ± 0.22^ab^	−7.00
MCV (fl)	71.23 ± 2.76^b^	73.33 ± 1.33^ab^	73.84 ± 1.68^ab^	75.24 ± 1.46^a^	5.60
MCH (pg)	27.38 ± 0.59	26.48 ± 0.47	26.43 ± 0.89	27.00 ± 0.87	−1.40
MCHC (g/dl)	36.42 ± 2.43	36.15 ± 1.30	37.15 ± 2.68	36.30 ± 2.53	−0.30
WBC (×10^9^/l)	12.86 ± 3.99^ab^	9.88 ± 2.84^b^	14.86 ± 2.76^ab^	17.84 ± 5.46^a^	38.70
Lymphocytes (%)	4.10 ± 1.32	4.15 ± 1.38	5.72 ± 2.49	6.75 ± 5.53	64.10

Note

a, b, c: means with different letters are significantly different at *p* < 0.05, ER, effect rate; bw, body weight; Hb, haemoglobin; PVC, packed cell volume; RBC, red blood cell; MCV, mean cell volume; MCHC, mean corpuscular haemoglobin concentration; MCH, mean corpuscular haemoglobin; WBC, white blood cells.

### 3.5 Biochemical parameters

The serum concentration of total cholesterol, creatinine, urea, ALAT, ASAT increased significantly (*p* < 0.05) in WILLOSATE‐treated groups relative to controls while serum total protein decreased significantly (*p* < 0.05) in a dose‐dependent manner compared to controls. There was no significant difference on glucose and serum total albumin in treated groups compared to controls (Table 5).

**TABLE 5:** Effects of varying doses of WILLOSATE on biochemical parameters in male guinea pig

Parameters (*n* = 10)	Dose of WILLOSATE (mg/kg bw)				
	0	186	280	560	ER (%)
Total cholesterol (mg/dl)	133.57 ± 9.70^b^	149.62 ± 15.30^b^	179.70 ± 19.79^ab^	184.07 ± 12.90^a^	37.80
Creatinine (mg/dl)	0.76 ± 0.08^ab^	0.73 ± 0.08^b^	0.83 ± 0.10^ab^	0.93 ± 0.14^a^	22.40
Urea (mg/dl)	81.71 ± 19.36^c^	166.28 ± 32.12^a^	121.29 ± 26.78^bc^	152.81 ± 29.04^ab^	87.10
ALAT (IU)	17.88 ± 1.96^b^	21.06 ± 3.85^b^	28.86 ± 1.48^a^	31.15 ± 1.57^a^	74.20
ASAT (IU)	18.64 ± 1.42^c^	22.56 ± 1.98^b^	34.91 ± 4.69^a^	35.18 ± 2.82^a^	88.70
Glucose (mg/dl)	29.54 ± 18.68	39.09 ± 17.61	26.59 ± 22.31	27.04 ± 12.17	−8.40
Total protein (g/dl)	2.37 ± 0.15^a^	2.36 ± 0.16^a^	2.08 ± 0.05^b^	2.09 ± 0.05^b^	−11.80
Total albumin (g/dl)	3.34 ± 0.17	3.32 ± 0.38	3.22 ± 0.22	3.14 ± 0.21	−5.90

Note

a, b, c means with different letters are significantly different at *p* < 0.05. ER, effect rate; bw, body weight; ALAT, alanine aminotransferase; ASAT, aspartate aminotransferase.

## 4 DISCUSSION

The results of this study showed a decrease in final body weight (−9.80%), body weight gain (−102.20%) and feed consumption (−13.10%) in male guinea pigs treated with increasing doses of WILLOSATE when compared to controls. The dose‐dependent decrease on body weight gain reported in the present study is in agreement with findings in mice exposed to 50 and 500 mg/kg body weight of ROUNDUP^®^ for 15 days (Jasper et al., 2012) and in Wistar rats exposed to pirimiphos‐methyl at 41.67, 62.5 and 125 mg/kg body weight for 90 days (Ngoula et al., 2007). The two herbicides used by these investigators are glyphosate formulations similar to GBH used in the present study. The negative effects of WILLOSATE on body weight performance may be ascribed to the demyelinating effects on the nervous system hence impairment of growth and development. Comparative studies on murine embryonic dorsal ganglionic cultures using pure glyphosate and glyphosate‐based herbicide reported a concentration‐dependent demyelinating effects of GBH and not pure glyphosate (Szepanowski et al., 2018). The cytotoxic effects of GBH could be ascribed to the composition of fomulants for the herbicides. Many pesticide manufacturing companies have been found to use petroleum distillates and polyoxyethylenamines (POEA) as well as heavy metals (Defarge et al., 2017), which may contribute to cytotoxicity effects on peripheral nervous system reported (Szepanowski et al., 2019). Fox (1999) and Ganong (2001) reported that stimulation of the central nervous system centres might cause an increase in food intake while a bilateral lesion of this area induces a complete cessation of food intake. According to Chahoud et al. (1999), weight loss is an important indicator of toxicity; thus, it can be inferred that WILLOSATE induced systemic toxicity, which may be associated with its capacity to stimulate reactive oxygen species (ROS) production.

Results of the present study showed a steady increase in relative weight and volume of liver (25.4%) and kidney (28.8%) and a decrease in testis weight (−24.0%) and volume (−32.0%) with increasing dose of WILLOSATE. Assessment of the toxic potential of a substance relies on the evaluation of weights, structure and function of detoxifying organs (liver and kidney) (Oloyede et al., 2011), which are responsible for drug metabolism and detoxification (Mossa et al., 2015). The increase in the weight of kidney and liver in animals exposed to WILLOSATE in this study is similar with the increase reported by Vemo et al. (2018) in male guinea pigs exposed to 92, 137.5 and 275 mg/kg b.w/day of Cypermethrin for 90 days and Djeffal (2014) in rats submitted to 8 mg/kg b.w/day of methomyl. This increase in the weight and volume of these organs could be due to the intensive activity of detoxification carried out by these organs. The histological structure of the kidney and liver were not considered in the present study to verify a possible alteration of function in these organs, and this can form basis for future investigation on this measure. However, increase in the serum level of urea (87.1%), creatinine (22.4%), ALAT (74.2%) and ASAT (88.7%) in the present study could be a consequence of functional and structural disturbance of the liver and kidneys as it has been reported elsewhere (Vemo et al., 2018). Abdel‐Wahhab et al. (2007) reported that the liver functional transaminases (ASAT and ALAT) enzymes activity in serum are indicators of liver diseases such as infectious hepatitis, alcoholic cirrhosis, biliary obstruction, toxic hepatitis and liver cancer. The significant increase in serum ASAT and ALAT in WILLOSATE‐ treated guinea pigs in the present study agrees with the findings of Benedetti et al. (2004) in male Wistar rats exposed to glyphosate‐Biocarb^®^ for 75 days. The increase in levels of these enzymes was explained by liver cellular alterations, with an increase in connective tissue and deposition of collagen in liver cells (Benedetti et al., 2004). The significant increase in serum urea and creatinine reported in the present study is similar to the results of Djeffal (2014) in rats exposed to 8mg/kg body weight/day of methomyl. This finding was ascribed to reduction in glomerular filtration in the kidney hence dysfunction of kidney tubules (Walmsley & White, 1994).

The relative weight of testis significantly decreased in WILLOSATE‐treated groups in a dose‐dependent manner compared to the control. Similar observations were made by Ngoula et al. (2007) in Wistar rats exposed to 41.67, 62.5 and 125 mg/kg body weight of pirimiphos‐methyl for 90 days. According to Gore (2001), the weight, size and the secretory function of testes are closely regulated by androgens, which are deemed to increase the mass of the testis. The decrease in the testicular weight could be explained by the fact that WILLOSATE had induced the alteration of the testicular structure hence interfered with spermatogenesis. The decrease in the sperm characteristics in WILLOSATE‐treated guinea pigs noticed in this study was previously reported by Owagboriaye et al. (2017) in male Albino rats orally exposed to 3.6, 50.4 and 248.4 mg/kg body weight of ROUNDUP® for 12 weeks. The decrease in sperm quality could be attributed to the disintegration of the plasma membrane following the over production of ROS (Agarwal et al., 2003), probably induced by WILLOSATE in the guinea pigs. In their study, it was reported that excessive generation of ROS in sperm by leukocytes as well as by abnormal spermatozoa could be a cause of the low sperm quality. The observed abnormal sperm cells in the present study could be attributed to chromosomal aberration (Piomboni et al., 2014), sperm mitochondrial DNA deletions (Talebi et al., 2017) or point mutation as previously reported (Narayana et al., 2002). Bruce and Heddle (1979) reported that the occurrence of sperm head abnormalities is attributed to the chromosomal aberrations that occur during the packaging of genetic material in the sperm head or occurrence of a point mutation in sperm cell mitochondrial DNA. Additionally, the decrease in the sperm characteristics in WILLOSATE‐ treated guinea pigs in the present study could have correlated positively with observed decrease in serum protein level (−11.8%), which confirms findings of earlier studies (Ngoula et al., 2007). Proteins are the most abundant macromolecules of cells of living organisms that contribute to the architecture and physiology of cells and cell metabolism (Mommsen & Walsh, 1992).

Results of the present study showed a significant decrease in RBCs and Hb by 7% and 5.7%, respectively, while WBC and MCV increased by 38.7% and 5.6%, respectively, in treatment groups compared to controls. Riaz and Yousafzai (2017) reported similar results in male rabbits exposed to Malathion and Cypermethrin at 75 mg/kg body weight for 7 days. White blood cells aid the body to fight infections and external noxious agents. Higher WBC count observed in the present study attested the deleterious effects of WILLOSATE on the blood parameters and portrayed an attempt by the body immune system to overcome the toxicants hence increase in number (Riaz & Yousafzai, 2017). The decreased RBC and Hb levels reported in this study is similar to the findings of Jasper et al. (2012) where mice treated with 500 mg/kg body weight of ROUNDUP® showed significant decrease in RBCs, Hb and hematocrit content with increase in MCV. The authors attributed these findings to the reduction in RBC count to presence of POEA in the formulants of glyphosate in the presence of few antioxidant defenses on cell membranes of RBCs that favoured formation of methemoglobin, lipoperoxidation and lysis of RBCs. Other studies have ascribed cytotoxicity caused by GBH to damage of cellular DNA owing to increased levels of ROS (Ortiz‐Ordoñez et al., 2011). Furthermore, WILLOSATE toxicity on RBC may cause hypoxia as the RBC highly serve transport function of blood gas carrying around 98% of oxygen throughout the body system (Jensen, 2009). The decrease in RBC may have led to the reduction in Hb reported in this study as the latter is the protein molecule in RBCs. It is also possible that substantial decrease in Hb resulted from suppression of erythropoiesis and heme synthesis by WILLOSATE as well as devastation of erythrocyte in hemopoietic tissue (Fetoui et al., 2008) or chromosomal aberrations in bone marrow cells (Prasad et al., 2009). Increased MCV has been also reported by Shah et al. (2007) in rabbits exposed to 25, 50 and 75 mg/kg of body weight of Cypermethrin. According to Barger (2003), the increase in MCV points towards macrocytic and hypochromic anaemia, probably due to the increased activity of bone marrow and deficiency of some hemopoietic factors previously mentioned above.

## 5 CONCLUSION

WILLOSATE had deleterious effects to male guinea pig after 60 days of treatment. For biochemical parameters, only serum total cholesterol and albumin were not affected while kidney and liver weights increased. The weight of testes was the only reproductive organ affected by the WILLOSATE dose. Growth performances, epididyemal sperm characteristics, weight of detoxifying organs and haematobiochemical parameters were seriously impaired. We can conclude that the deleterious effects observed in the present study may be attributed to the oxidative stress caused by the formulations in the WILLOSATE. Finally, it is recommended that the use of WILLOSATE even at low dose (186 mg/kg body weight) must be limited due to its hazardous effect on animals and human since they have quite similar physiology. For future studies it will be necessary to isolate individual components of WILLOSATE and evaluate their toxicity effects.

## 6 ANIMAL WELFARE STATEMENT

The authors confirm that the ethical policies of the journal, as noted on the journal's author guidelines page, have been adhered to and the appropriate ethical review committee approval has been received. The authors confirm that they have followed EU standards for the protection of animals used for scientific purposes [and feed legislation, if appropriate].

## 7 STUDY SITE, ANIMAL HOUSING AND FEEDING

The study was conducted at the experimental unit of the Department of Animal Production of the Université Evangélique en Afrique (UEA) in Bukavu city, DR Congo. All laboratory work was conducted in the laboratory of Animal Physiology of the UEA, Faculty of Agriculture and Environmental studies. Forty (40) local sexually mature male guinea‐pigs weighing 397.89 ± 38.84 g were raised at the farm of the Faculty of Agriculture and Environmental Studies of UEA.

## CONFLICT OF INTEREST

The authors declare no competing interests.

## AUTHOR CONTRIBUTION

Valence Bwana Mutwedu, Daniel Katulanya Chiregereza, Rodrigue Basengere Balthazar Ayagirwe participated in the design of the experiment, supervised the experiment and drafted the manuscript. Valence Bwana Mutwedu, Daniel Katulanya Chiregereza, Rodrigue Basengere Balthazar Ayagirwe participated in conducting the experiment. Albert Wafula Nyongesa, Pascaline Ciza Azine, Victor Herman Ngoumtsop and Yannick Mugumaarhahama helped draft the manuscript and provided mentorship support.

**Valence Mutwedu:** Conceptualization; Formal analysis; Methodology; Writing‐original draft; Writing‐review & editing. **Albert Nyongesa:** Formal analysis; Writing‐original draft; Writing‐review & editing. **Azine Ciza:** Data curation; Formal analysis; Methodology; Writing‐original draft; Writing‐review & editing. **Daniel Chiregereza:** Conceptualization; Methodology; Writing‐original draft. **Herman Ngoumtsop:** Validation; Writing‐original draft; Writing‐review & editing. **Yannick Mugumaarhahama:** Data curation; Formal analysis; Writing‐original draft; Writing‐review & editing. **Rodrigue Ayagirwe:** Conceptualization; Data curation; Methodology; Software; Supervision; Writing‐original draft; Writing‐review & editing.

## ETHICAL STATEMENT

The experimental protocol was approved by the Ethical Committee of the Université Evangélique en Afrique, and the experiments were performed in accordance with the internationally accepted standard ethical guidelines on protection of animals used for scientific purposes as described in the European Community guidelines; EEC Directive 2010/63/EU of 1st January 2013.

### Peer Review

The peer review history for this article is available at https://publons.com/publon/10.1002/vms3.443.
